# Genetic transformation of the oilseed crop camelina using immature zygotic embryos

**DOI:** 10.1186/s13007-025-01351-2

**Published:** 2025-03-13

**Authors:** Barno Ruzimurodovna Rezaeva, Ingrid Otto, Götz Hensel, Pouneh Pouramini, Anton Peterson, Jochen Kumlehn

**Affiliations:** 1 Plant Reproductive Biology, Leibniz Institute for Plant Genetics and Crop Plant Research (IPK), Corrensstrasse 3, 06466 Seeland, Germany; 2 Centre for Plant Genome Engineering (CPGE), Heinrich Heine University Düsseldorf, 40204 Düsseldorf, Germany; 3 Cryo and Stress Biology, Leibniz Institute for Plant Genetics and Crop Plant Research (IPK), Corrensstrasse 3, 06466 Seeland, Germany

**Keywords:** Adventitious shoots, *Agrobacterium*, Genetic engineering, Pre-cultivation, Wound response, Transgenic plants

## Abstract

**Background:**

Camelina is an oilseed crop with particularly useful fatty acid and amino acid profiles of its seeds, high resilience to abiotic and biotic stresses, and a short life cycle. Previous genetic engineering approaches in camelina have largely relied on the floral dip method which is, however, associated with genotype-dependent efficiency and incompatibility with methods of direct biomolecule delivery.

**Results:**

Here, we established a novel method of transgenesis for camelina, taking advantage of the high regenerative capacity of immature embryos. Various culture conditions and treatments were experimentally validated, which included the duration of explant pre-cultivation, wounding of explants and its time of application, *Agrobacterium* strain and density of inoculum, acetosyringone concentration, duration of explant-*Agrobacterium* co-cultivation, as well as application time and concentration of the selective agent hygromycin. We provide convergent evidence of stable transgenicity and transgene inheritance by (1) selection for resistance to hygromycin, (2) PCR, (3) detection of the transgene product GFP, and (4) DNA gel blot analysis involving primary transgenic plants and segregating progeny. Primary transgenics examined in detail featured one to three T-DNA integration loci, with one to seven T-DNA copies being integrated in total per plant. The established method proved efficient across all three tested accessions including two current cultivars, whereby transformation efficiencies, determined as PCR-positive primary transgenic plants related to agro-inoculated explants, of between 13 and 17% were obtained.

**Conclusion:**

With this method, we provide a viable platform for the functional validation of genes-of-interest and for biotechnological improvements of plant performance and quality features in camelina.

## Background

Camelina (*Camelina sativa* (L.) Crantz) is an oilseed plant that, in comparison to, e.g., oilseed rape, better tolerates various adverse conditions, such as low temperatures, drought, and nutrient-poor soils. It requires only 85 to 100 days from planting to maturity, which allows for its use for intercropping. Thanks to the short life cycle, camelina has also been used as an experimental model plant for agricultural oilseed crops [1]. Camelina seeds feature a unique oil profile with a very high level of α-linolenic acid, which makes them a valuable source of the essential ω-3 fatty acids that have beneficial effects for human nutrition and are highly useful for the chemical industry too. On the other hand, the content of the undesired erucic acid is below 3% [2]. The press cake obtained during oil extraction is rich in proteins and can be used for animal feed, which further increases the economic value of camelina [3].

*Agrobacterium*-mediated transformation is often preferred over methods of direct DNA-transfer due to the comparatively low transgene copy numbers obtained, which is associated with a reduced risk of transgene silencing [4]. Over the past two decades, the *Agrobacterium*-based floral dip method has been broadly used to produce transgenic plants in camelina [5]. However, the transformation efficiency remained comparatively low and dependent on suitable and stable environmental conditions during plant cultivation. While the transformation of various camelina accessions has been shown via floral dip in some studies, this method still appears to have a considerable genotype dependency [6]. Apart from the few plant species for which the floral dip method can be employed, the genetic transformation of plants is usually carried out using cell and tissue cultures. In the case of camelina, however, no viable methods have yet been established on this basis. Yemets et al. [7] used cotyledons, petioles, and hypocotyl segments of seedlings for the inoculation with *Agrobacterium*. Later approaches involved camelina leave explants [8] or micropropagated shoot meristems [9]. However, none of these studies provided molecular evidence of genomic integration and inheritance of T-DNAs nor have they been later used in any applied approaches.

In the present investigation, a recently established method of adventitious shoot formation from immature zygotic embryos was employed [10] to develop a novel and robust method of *Agrobacterium*-mediated transformation in camelina. A comprehensive validation of treatments and culture conditions by comparative experiments using the accession Cam139 led to some methodological improvements. In addition to comprehensive analyses of transgenicity and its inheritability, the method was also validated with regards to genotype dependency, whereby a direct experimental comparison of Cam139 with the current cultivars Ligena and Calena resulted in consistently useful transformation efficiencies.

## Results

### Establishment of an *Agrobacterium*-based transformation protocol using camelina immature embryos

**Fig. 1:**
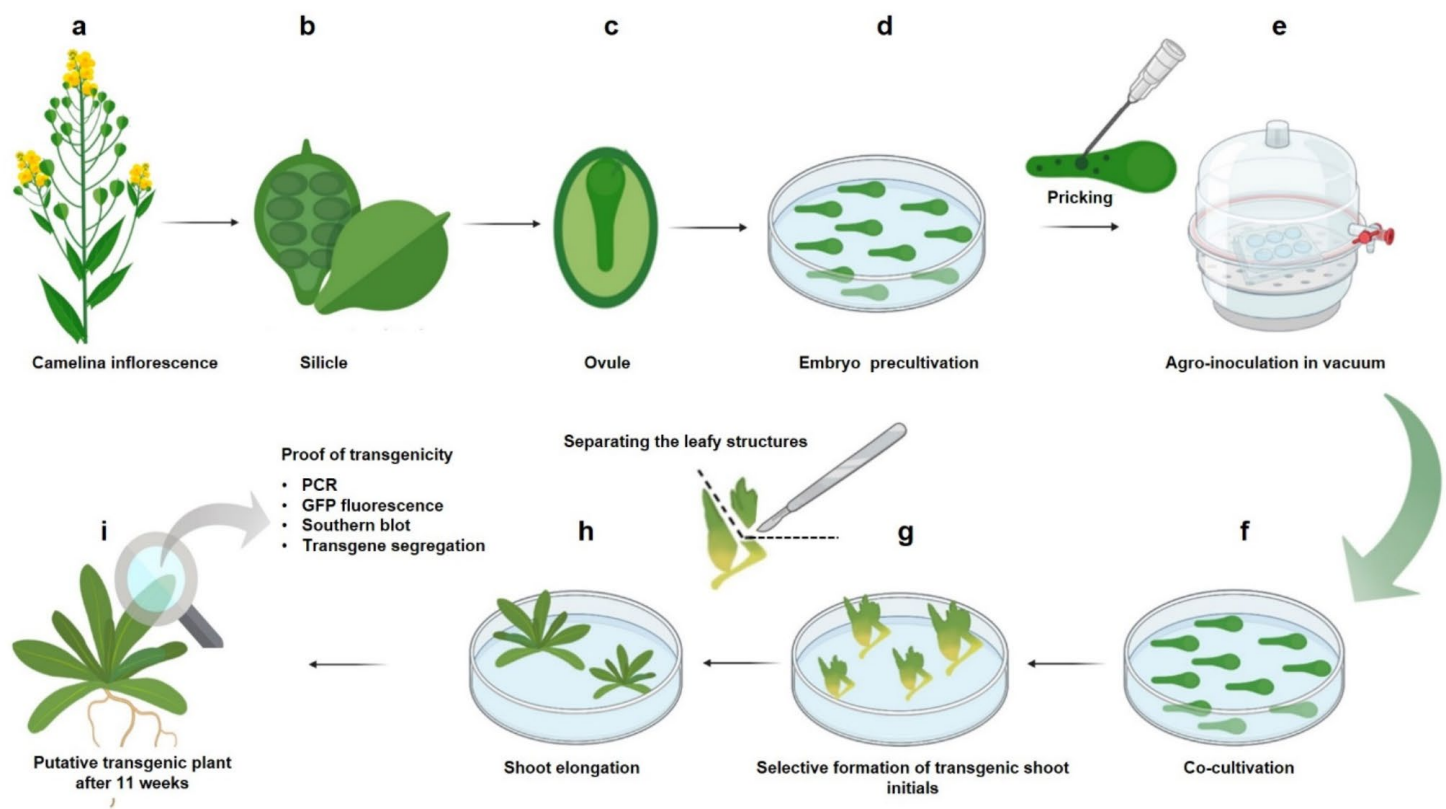
Schematic representation of *Agrobacterium*-mediated transformation of camelina using immature embryos. (**a**) Inflorescence with silicles after two weeks of pollination, (**b**) silicle with ovules, (**c**) isolated ovule with immature embryo, (**d**) pre-cultivation of embryos, (**e**) agro-inoculation in a vacuum bell jar right after wounding of embryo hypocotyls, (**f**) co-cultivation of embryos with *Agrobacterium*, (**g**) induction of neoplastic development and formation of adventitious shoot initials under selective conditions, (**h**) shoot elongation, (**i**) plantlet formation followed by examinations regarding transgenicity. Created by Procreate and Biorender (biorender.com)

The workflow of the established procedure is shown in Fig. 1. The process starts with the collection of donor plants’ inflorescences two weeks after pollination. Immature zygotic embryos are then excised from the silicles and precultivated. Before vacuum-assisted agro-inoculation, the embryo explants were pricked to facilitate entry of *Agrobacterium* and wound-induced formation of regenerable structures. After inoculation, the explants are co-cultivated to allow *Agrobacterium* to transform infected cells. Within the following three month, adventitious shoots and plantlets are formed under selective conditions that ensure preferential development of transgenic tissue. Finally, transgenic plants are confirmed at the molecular level, detection of reporter-derived fluorescence and segregation analysis.

The presentation of results corresponds to the chronology of the established protocol rather than the order in which they were conducted. The successively started experiments had a considerable temporal overlap with each other, which is why an immediate implementation of findings in subsequent experiments was only possible in exceptional cases.

### Combined pricking and agro-inoculation stimulate adventitious shoot formation from immature zygotic embryos

To our surprise, preliminary experiments had indicated that the formation of adventitious shoots was increased in some transformation experiments as compared to non-transformed control cultures. To gain further insight into this phenomenon, the effects on adventitious shoot formation of pricking, agro-inoculation or the combination of both in comparison to no such treatments were investigated. In this experiment, the plant selective agent hygromycin was generally omitted to avoid compromising the treatments without agro-inoculation, in which no hygromycin-resistant shoots could develop. In contrast, the antibacterial agent Timentin was used in all treatments to ensure the same basic circumstances on the one hand and to effectively suppress bacterial development in the treatments that included agro-inoculation on the other.

The data presented in Table 1 were recorded after 10 weeks of explant cultivation. The experiment shows that the combination of pricking and agro-inoculation leads to an about five-fold increase in adventitious shoot formation compared with the untreated control, while pricking alone still results in about a doubling of efficiency. Agro-inoculation without pricking, on the other hand, showed no such effect.

**Table 1:** Effect of pricking and agro-inoculation on the formation of adventitious shoots from immature embryo explants

No. of IZEs^1^	Treatment	No. of adventitious shoots formed^2^
60	no (control)	30^c^
60	pricking	66^b^
60	agro-inoculation	30^c^
60	pricking + agro-inoculation	161^a^

^1^20 immature zygotic embryos (IZEs) of line Cam139 were cultivated per culture dish, with three dishes being used as repetitions per treatment

^2^Data were subjected to Normality and Equal Variance tests followed by Student’s t-test. If a pair of treatments did not comply with equal variance, Welch’s t-test was applied. Different superscript letters given for two treatments denote a significant difference (*P* ≤ 0.05)

### Effect of pre-cultivation period and time point of pricking

To identify particularly suitable conditions for efficient transformation, the duration of pre-cultivation and the time point of pricking was varied and compared. In addition to the variables and treatments specified in Table 2 and in contrast to the standard procedure described in the Materials and Methods section, the conditions of this experiment involved selection using 25 mg/L hygromycin for 4 weeks. Even though the differences in transformation efficiency were not significant in this experiment, it is worth noticing that pricking right before agro-inoculation consistently resulted in higher numbers of GFP-positive plants per 60 explants than pricking one day before agro-inoculation. In addition, pre-cultivation for 3 days before agro-inoculation resulted in a higher number of transgenic plants than two days of pre-cultivation, provided wounding was conducted right before inoculation.

**Table 2:** Effect of pre-cultivation period and time point of pricking of immature zygotic embryos on transformation efficiency

No. of IZEs^1^	Duration of pre-cultivation (before inoculation)	Time point of pricking	No. of regenerated plantlets	No. of PCR- positive plants and efficiency^2^
60	2 days	after 1 day of pre-cultivation	6	3 (5%)
60	2 days	after 2 days of pre-cultivation	14	5 (8%)
60	3 days	after 2 days of pre-cultivation	8	2 (3%)
60	3 days	after 3 days of pre-cultivation	22	7 (12%)
60	3 days (control without inoculation)	after 3 days of pre-cultivation	0	0 (0%)

^1^20 immature zygotic embryos (IZEs) of line Cam139 were cultivated per culture dish, with three dishes being used as repetitions per treatment

^2^PCR was performed using primers specifically amplifying the *GFP* transgene, and the transformation efficiency was calculated as proportion of agro-inoculated explants giving rise to *GFP*-positive plants. Data were subjected to Kruskal-Wallis analysis of variance on ranks followed by all pairwise multiple comparison of treatments using the Student-Newman-Keuls method. The differences in transgenic plant formation between the treatments were not statistically significant (*P* ≤ 0.05)

### Effects of *Agrobacterium* density, acetosyringone concentration and duration of co-cultivation

To improve the transformation method, further conditions were validated in the context of agro-inoculation and co-cultivation of explants with *Agrobacterium*. The experimental variables and treatments are specified in Table 3.

**Table 3:** Effect of *Agrobacterium* density, duration of co-cultivation, and Acetosyringone concentration on transformation efficiency

No. of IZEs^1^	OD_600_ of Agrobacterium at inoculation	Duration of co-cultivation	Acetosyringone concentration [µM]	No. of regenerated plantlets	No. of PCR- positive plants and efficiency^2^
60	0.3	48 h	150	22	5^ab^ (8%)
60	0.3	48 h	300	27	4^b^ (7%)
60	0.3	72 h	150	37	7^ab^ (12%)
60	0.6	48 h	150	28	8^a^ (13%)
60	0.6	48 h	300	41	6^ab^ (10%)

^1^20 immature zygotic embryos (IZEs) of line Cam139 were cultivated per culture dish, with three dishes being used as repetitions per treatment

^2^PCR was conducted using primers specifically amplifying the *GFP* transgene, and the transformation efficiency was calculated as *GFP*-positive plants per number of agro-inoculated explants. Data were subjected to Normality and Equal Variance tests followed by comparison of treatments by Student’s t-test. Exclusively different superscript letters given for two treatments denote a significant difference (*P* ≤ 0.05)

Using the lower OD_600_ of 0.3, co-cultivation for 3 days resulted in higher number of transgenic plants than 2 days of co-cultivation. However, the highest number of transgenic plants was obtained using an agrobacterial density of OD_600_ of 0.6, 2 days of co-cultivation and 150 µM acetosyringone. However, a significant difference was only found between this combination of conditions in comparison to the treatment that has led to the poorest results in this experiment (Table 3).

### Comparison of two *Agrobacterium* strains

In this experiment, the hypervirulent *Agrobacterium* strains LBA4404/pSB1 and AGL1 were compared (Table 4). Strain AGL1 resulted in a significantly higher transformation efficiency as compared to LBA4404/pSB1. This was not only due to the more efficient plant regeneration, but also to a higher proportion of transgenics among the regenerated plants.

**Table 4:** Effect of *Agrobacterium* strains on the transformation efficiency

No. of IZEs^1^	Agrobacterium strain	No. of plantlets generated	No. of PCR-positive plants and efficiency^2^	% PCR-negative plants
60	LBA4404/pSB1	22	7b (12%)	68.2
60	AGL1	27	13a (22%)	51.9

^1^20 immature zygotic embryos (IZEs) of line Cam139 were cultivated per culture dish, with three dishes being used as repetitions per treatment

^2^PCR was performed using primers specifically amplifying the *GFP* transgene, and the transformation efficiency was calculated as proportion of agro-inoculated explants giving rise to *GFP*-positive plants. Data were subjected to Kruskal-Wallis analysis of variance on ranks followed by comparison of treatments using Student-Newman-Keuls method. Different superscript letters given for the two treatments denote a significant difference (*P* ≤ 0.05)

### Optimization of selective conditions during the formation of transgenic plants

For optimization of the selective conditions during shoot formation, the administration of hygromycin was varied in terms of application phase and concentration. The experimental treatments are specified in Table 5.

**Table 5:** Effect of hygromycin concentration and period of selection on transformation efficiency

No. of IZEs^1^	Hygromycin concentration (mg/L)				No. of plantlets generated	No. of PCR-positive plants and efficiency^2^
	Shoot initiation I	Shoot initiation II	Shoot elongation	Rooting		
60	25	0	0	0	22	4^ab^ (7%)
60	25	25	0	0	16	5^a^ (8%)
60	25	25	25	25	5	0^b^ (0%)
60	50	25	0	0	11	3^ab^ (5%)
60	50	25	25	25	8	2^ab^ (3%)
60	100	25	25	25	1	0^b^ (0%)

^1^20 immature zygotic embryos (IZEs) of line Cam139 were cultivated per culture dish, with 3 dishes being used as repetitions per treatment

^2^PCR was performed using primers specifically amplifying the *GFP* transgene, and the transformation efficiency was calculated as *GFP*-positive plants per number of agro-inoculated explants. Data were subjected to Normality and Equal Variance tests followed by all pairwise multiple comparison of treatments by Tukey test. Exclusively different superscript letters given for two treatments denote a significant difference (*P* ≤ 0.05)

**Fig. 2:**
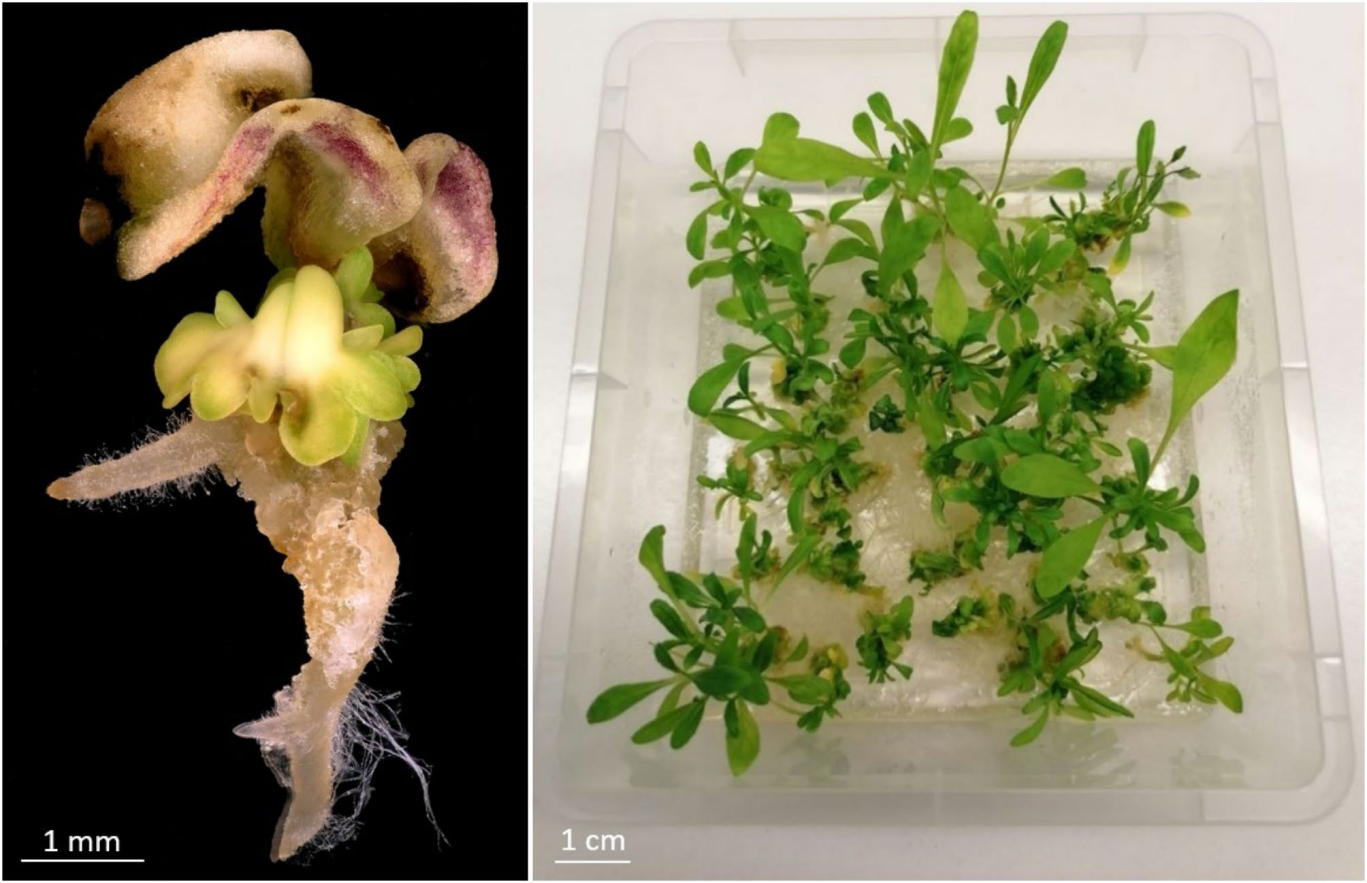
Formation of somatic embryos (left) and adventitious shoots (right) from immature zygotic embryo explants of line Cam139 after inoculation using *Agrobacterium* strain LBA4404/pSB1 and growth on shoot induction medium containing 25 mg/L hygromycin

The highest transformation efficiency (transgenic plants related to number of explants) was achieved in this experiment, when hygromycin was administered at 25 mg/L for a period of 4 weeks after co-cultivation. Under this condition, also the proportion of explants giving rise to non-transgenic regenerants (68.8%) was the lowest among those treatments through which transgenic plants were obtained. The formation of regenerable structures and plant regeneration under selective conditions is shown in Fig. 2.

### Suitability of the transformation method for different genotypes

In addition to line Cam139, the cultivars Ligena and Calena were used to examine the dependence of the established transformation method on the genotype. This test was conducted across two selection regimes, namely 25 mg/L hygromycin during the 4 weeks of shoot initiation versus 50 mg/L during the first 2 weeks and 25 mg/L during the following 2 weeks. The experimental variables and treatments are specified in Table 6. In contrast to the previous experiment, where a larger variety of selection regimes were compared, the higher hygromycin concentration (50 mg/L) during the first two weeks of the shoot initiation phase I resulted in the highest transformation efficiencies. This was not only seen in line Cam139, but also applied for the two cultivars Calena and Ligena. However, these differences were not statistically significant. All three genotypes tested were largely on a par with respect to transformation efficiency, with the highest number of transgenics being obtained in cv. Ligena. However, under both selective conditions and across all three genotypes, the proportion of non-transgenic survivors was rather high at over 70%.

**Table 6:** Test of the transformation method across three camelina genotypes

No. of IZEs^1^	Genotype	Hygromycin concentration [mg/L]		No. of plantlets generated	No. of PCR-positive plants and efficiency^2^	% PCR-negative plants
		Shoot initiation I	Shoot initiation II			
60	Cam139	25	25	27	4 (7%)	85.2
60	Cam139	50	25	37	9 (15%)	75.7
60	Calena	25	25	29	5 (8%)	82.8
60	Calena	50	25	76	8 (13%)	89.5
60	Ligena	25	25	22	6 (10%)	72.7
60	Ligena	50	25	53	10 (17%)	81.1

^1^20 immature zygotic embryos (IZEs) were cultivated per culture dish, with 3 dishes being used as repetitions per treatment

^2^PCR was performed using primers specifically amplifying the *GFP* transgene, and the transformation efficiency was calculated as *GFP*-positive plants per number of agro-inoculated explants. Data were subjected to Kruskal-Wallis analysis of analysis of variance on ranks followed by all pairwise multiple comparison of treatments using the Student-Newman-Keuls method. However, the differences in transgenic plant formation between the treatments were not statistically significant (*P* ≤ 0.05)

### Analysis of transgenic plants

#### Establishment of selective conditions for seed germination

**Fig. 3:**
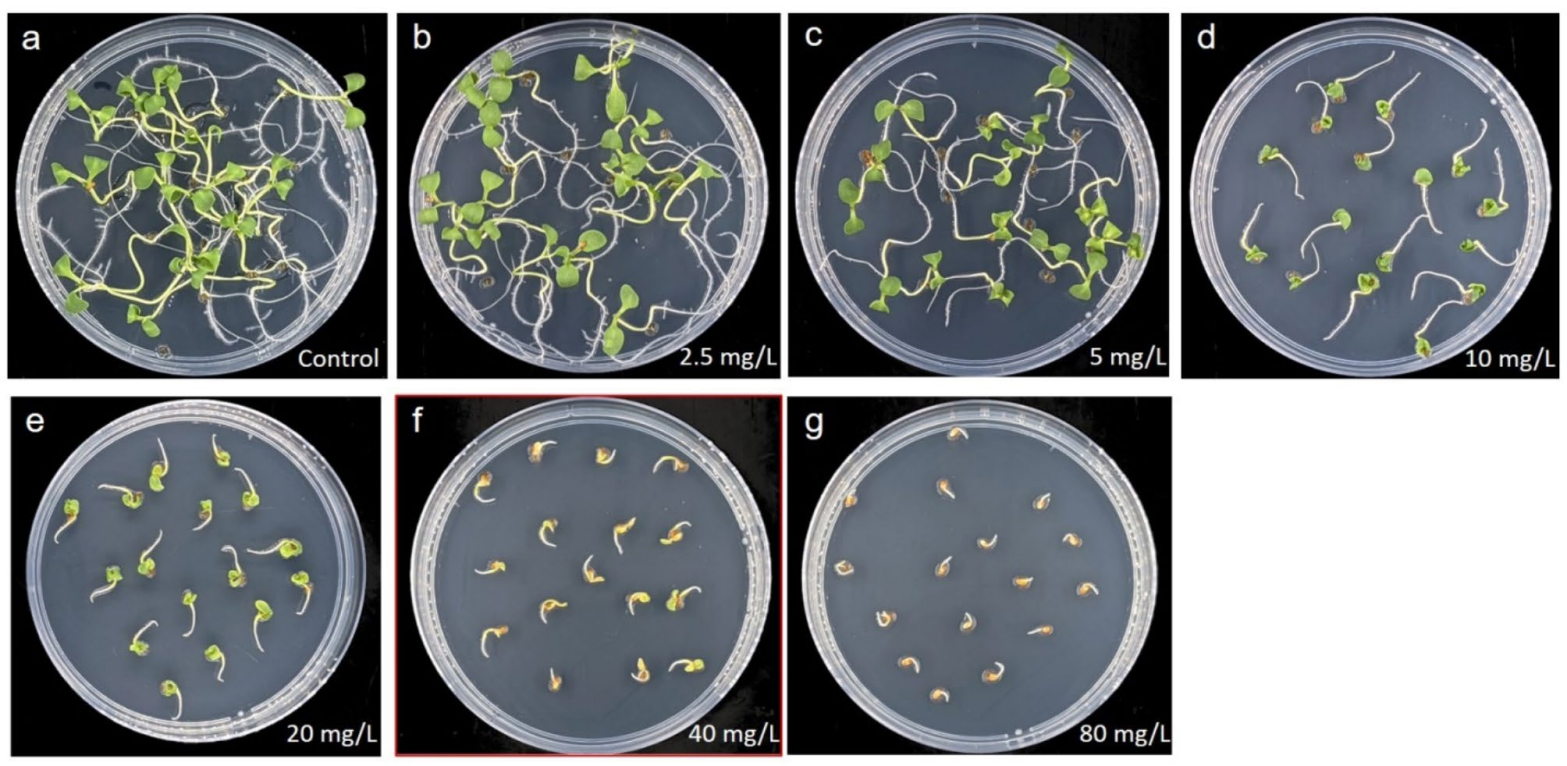
Effect of different hygromycin concentrations on germination in cv. Ligena using wild-type seeds. The images were taken 7 days after the seeds had been placed on germination medium. **A**) The medium used for the control treatment did not contain hygromycin. **B**-**G**) The hygromycin concentrations applied under the various selective conditions are indicated. 40 mg/L (edged in red) were used for following segregation analyses

To establish selective conditions that allow differentiation between hygromycin-resistant and hygromycin-sensitive seedlings in segregation analyses, wild-type seeds were placed on germination medium with different hygromycin concentrations. A clearly inhibited development of the seedlings was seen in the treatments with 10 mg/L or more, whereby even at the highest tested concentration of 80 mg/L hygromycin, all seeds still formed a short radicle while no primary shoot was emerging (Fig. 3). According to this observation, the formation of a radicle can be used as an indicator for the general germination capability of each individual seed. Based on these results, 40 mg/L hygromycin was used for subsequent segregation analyses of T_1_ families.

#### Evidence of generational transmission of transgenes and their segregation in progeny

**Fig. 4:**
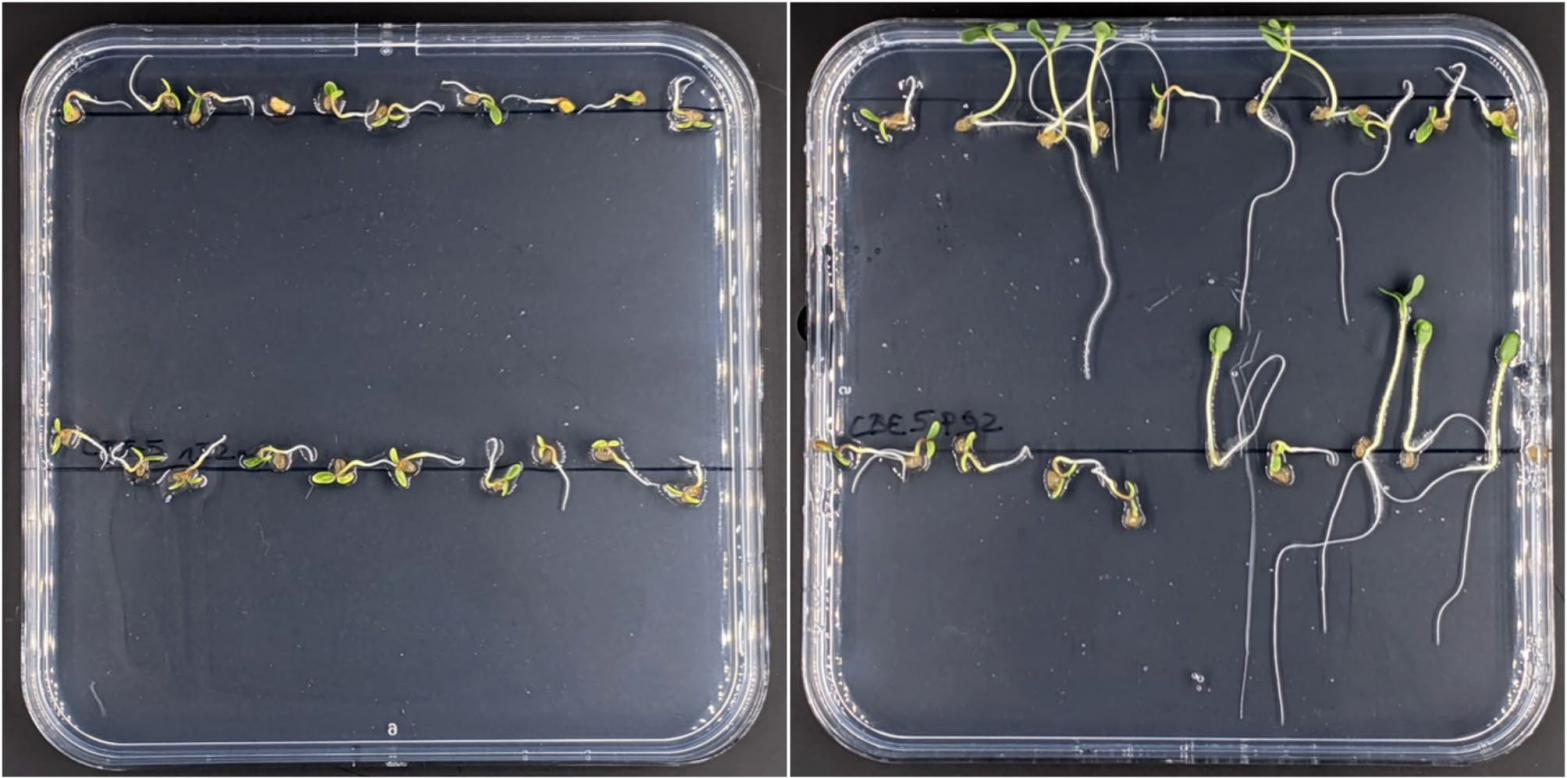
Analysis of transgene segregation in T_1_ siblings of cv. Ligena in 12 × 12 cm plates. The germination medium was supplemented with 40 mg/L hygromycin resulting in selective development of *hpt*-transgenic individuals. Whereas the non-transgenic control seeds on the left plate generally discontinued germination after emergence of the radicle, some of the T_1_ siblings derived from the primary transgenic plant P92 and grown on the right plate proofed unaffected by the selective agent hygromycin. The pictures were taken after four days of germination

**Fig. 5:**
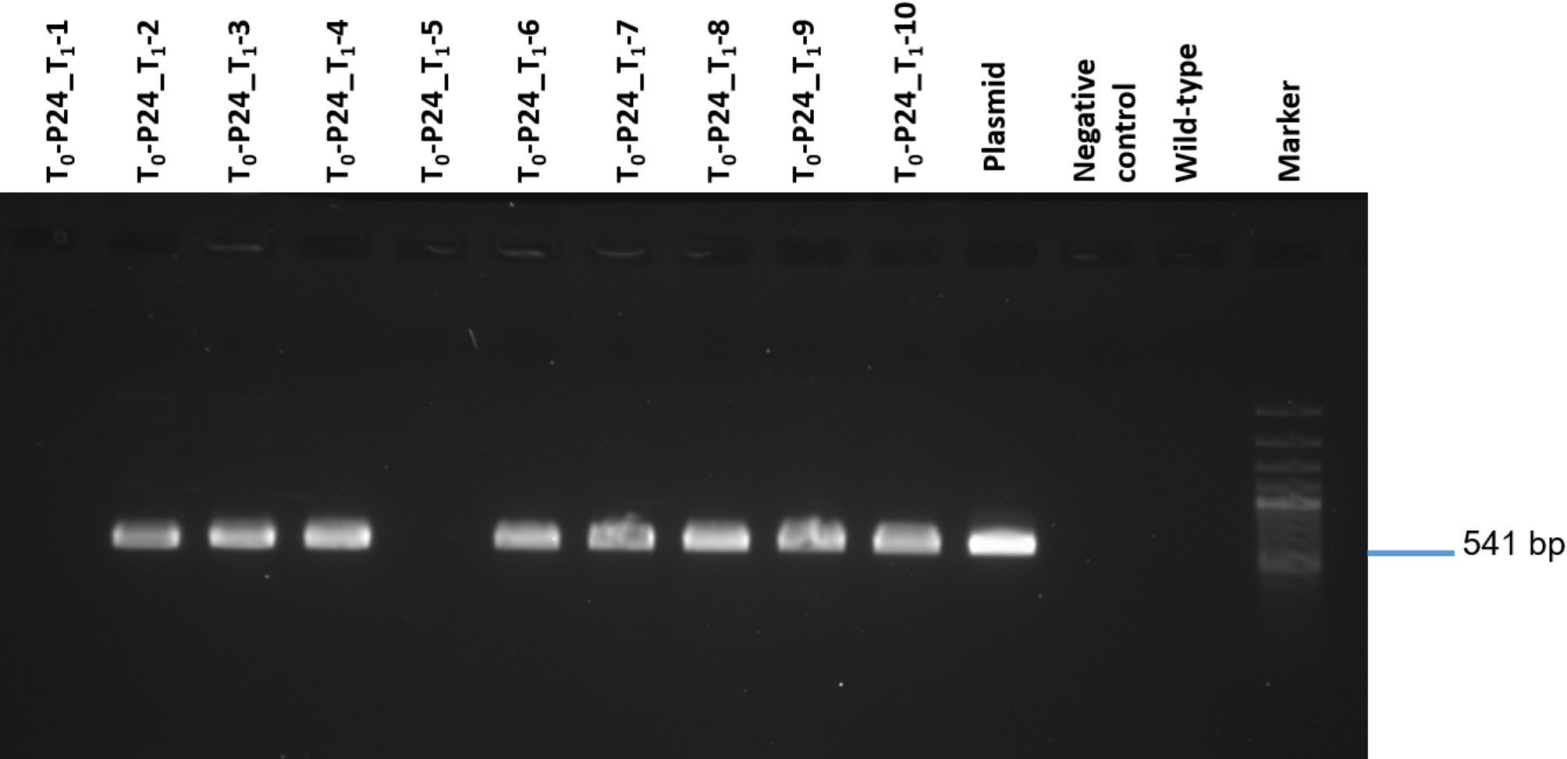
Confirmation of putative T_1_ transgenic plants by PCR using *GFP*-specific primers. Lanes 1–10: PCR-amplicons of randomly chosen T_1_ siblings derived from primary transgenic plant P24 of cv. Calena. Like in the wild-type (WT) control plant, no *GFP*-specific amplicon was obtained from plants T_1_-1 and T_1_-5 indicating them to be null segregants, whereas all other tested T_1_ siblings proved PCR-positive indicating their transgenicity. P: plasmid DNA, N: negative control (Master Mix without DNA), M: 100 bp DNA Ladder (Thermo Fisher Scientific), the expected position for 541 bp *GFP*-specific amplicons is indicated

**Fig. 6:**
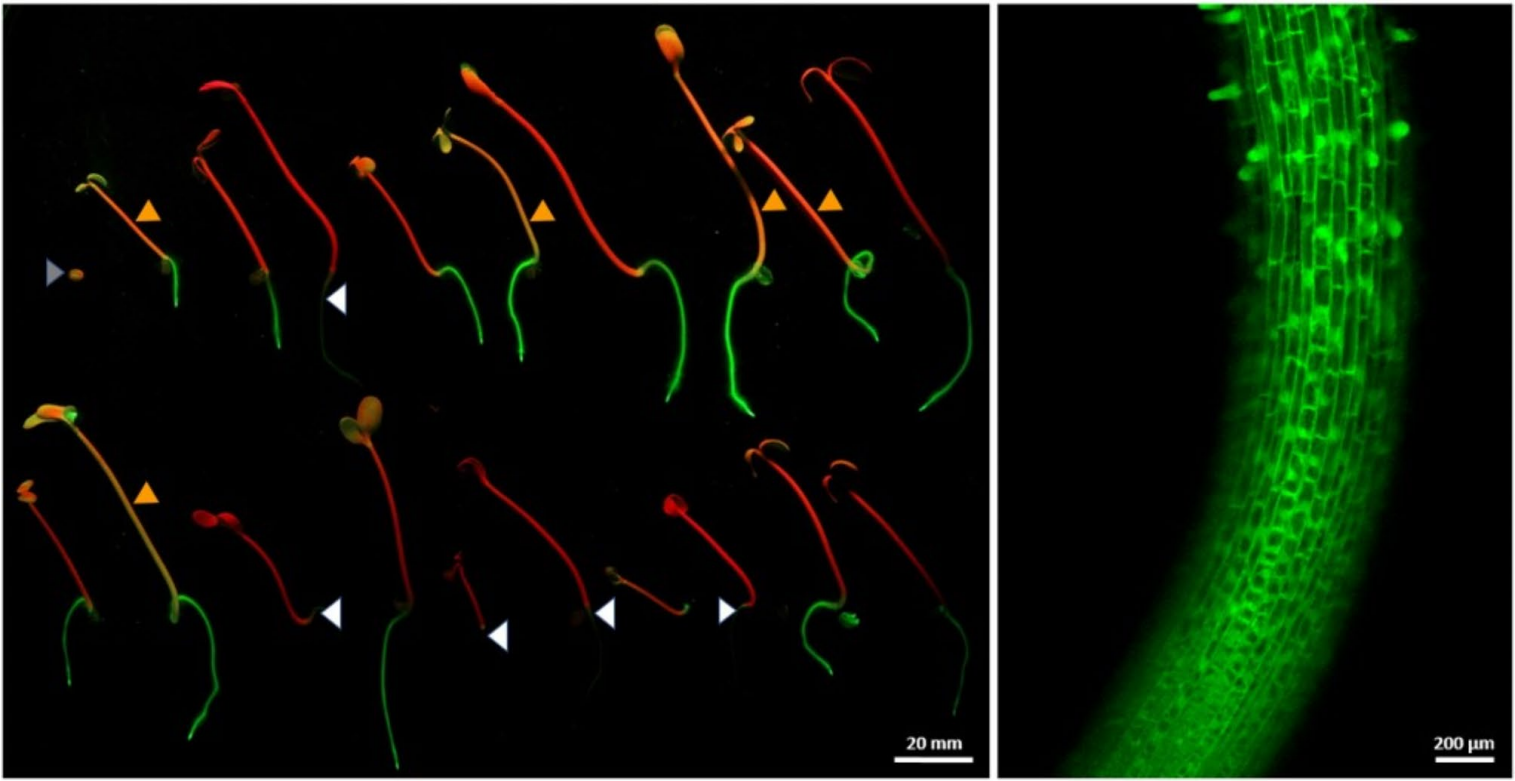
Evaluation of transgene expression and segregation of T_1_ siblings derived from cv. Ligena T_0_ plant P5 that carried a constitutively expressed *GFP* gene in hemizygous condition. Left: Out of the 20 randomly chosen T_1_ siblings shown, five null segregants show bright red fluorescence in their above-ground parts due to the chlorophyll content, while their roots are hardly visible (white arrowheads) owing to the missing *GFP* gene. By contrast, the 14 *GFP*-transgenic siblings show bright green fluorescence in their roots, with the hypocotyls and cotyledons of particularly strong *GFP*-expressors (possibly homozygous transgenic segregants) appearing in orange color (orange arrowheads) owing to the mixture of light emitted by chlorophyll (red) and GFP (green). One seed (upper row, far left, grey arrowhead) did not germinate and hence was not considered in the segregation analysis. The image was taken by a Canon EOS M200 camera. Right: Representative image of the root elongation zone of a transgenic sibling captured by confocal laser scanning microscopy. Both images were taken after four days of germination upon excitation by monochromatic light at 488 nm and the use of GFP-specific filter sets

**Fig. 7:**
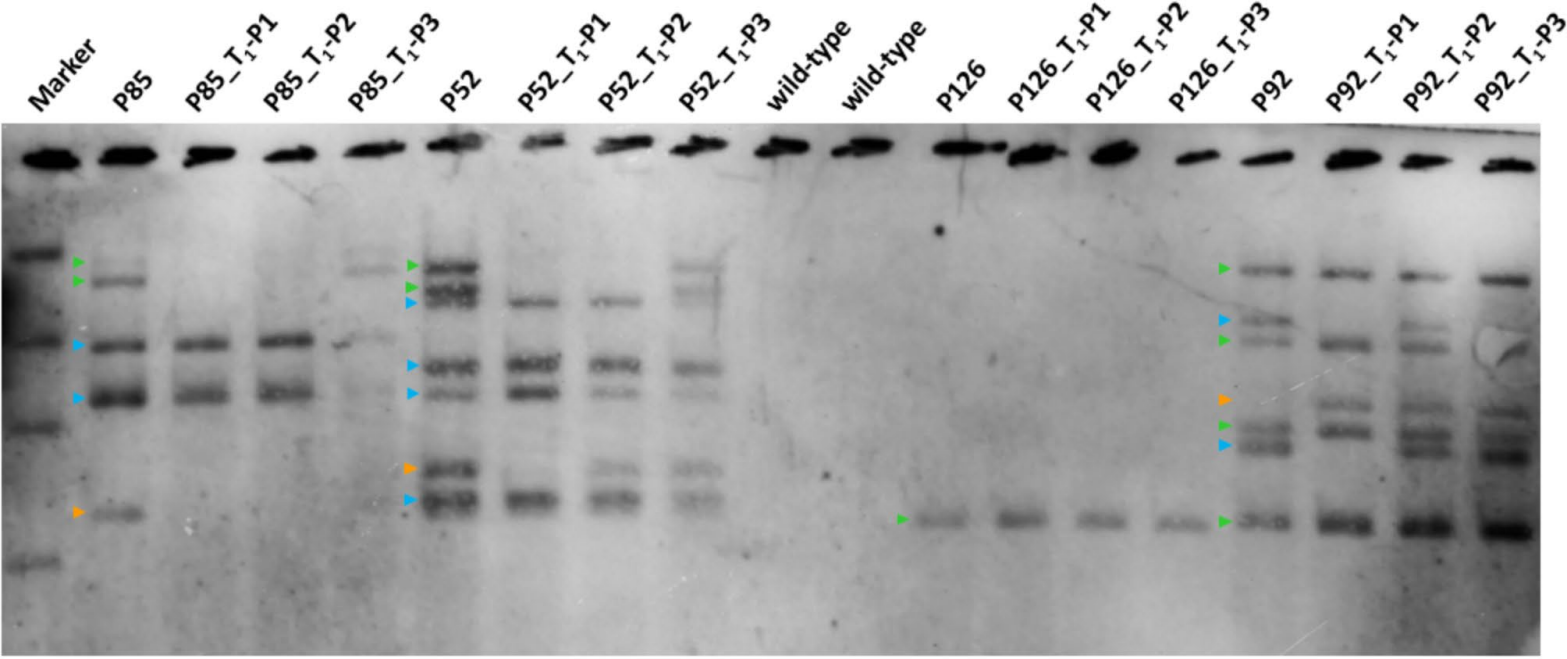
DNA gel blot analysis of four primary transgenic plants and three transgenic progeny each that segregate for different integration sites. Plant genomic DNA was digested with HinDIII and the fragments were separated in a 0.8% (w/v) agarose gel. T-DNA-containing fragments were visualized upon hybridization with a *GFP-*specific DNA probe. Two wild-type plants were used as negative control. Groups of genetically coupled T-DNA copies deemed to be integrated in the same genomic site are indicated by arrowheads of the same color. The color code was independently applied to each of the four families of transgenic plants analyzed

The transgenicity and its segregation in the T_1_ generation was determined by *hpt*-conferred hygromycin resistance (Fig. 4; Table 7), by *GFP*-specific PCR (Fig. 5; Table 8), by examination of GFP fluorescence in the plants (Fig. 6; Table 9), and by DNA gel blot analysis (Fig. 7; Table 10).

**Table 7:** Analysis of transgene segregation in T_1_ siblings of Cv. Ligena via germination on medium supplemented with 40 mg/l hygromycin

T_0_ mother plants	Number of T_1_ plants analyzed	Hygromycin-resistant vs. susceptible seedlings	Segregation observed (expected)	χ^2^ value
P5	20	11:9	1.2:1 (3:1)	4.3
P52	140	26:114	1:4.4 (3:1)	237.7
P85	40	28:12	2.3:1 (3:1)	0.53*
P126	60	25:35	1:1.4 (3:1)	35.6

The formation of a radicle indicated germination ability of all seeds analyzed. Hygromycin-susceptible (non-transgenic) seedlings ceased their development after the emergence of the radicle

*The asterisk indicates that the segregation obtained does not significantly deviate from the expected (Mendelian) ratio according to the *Chi*-square test at the significance level of 0.05

**Table 8:** Analysis of transgene segregation in T_1_ siblings of Cv. Calena using *GFP*-specific PCR

T_0_ mother plants	Number of T_1_ siblings analyzed	Seedlings with GFP-positive vs. negative PCR	Segregation observed (expected)	χ^2^ value
P8	20	16:4	4:1 (3:1)	0.27*
P9a^1^	20	10:10	1:1 (3:1)	6.67
P9e	20	13:7	1.86:1 (3:1)	1.07*
P21a	20	16:4	4:1 (3:1)	0.27*
P24	20	4:16	1:4 (3:1)	32.27

^1^Smaller case letters included in the plant code specify different sister lines derived from the same embryo explant

*Asterisks indicate that the segregation obtained does not significantly deviate from the expected (Mendelian) ratio according to the *Chi*-square test at the significance level of 0.05

**Table 9:** Analysis of transgene segregation in T_1_ siblings of Cv. Ligena based on GFP fluorescence via germination on hygromycin-free medium

T_0_ mother plants	No. of T_1_ seedlings analyzed	GFP fluorescence-positive vs. negative seedlings	Segregation observed (expected)	χ^2^ value
P5	19	14:5	2.8:1 (3:1)	0.92*
P52	20	6:14	1:2.3 (3:1)	21.6
P85	20	13:7	1.86:1 (3:1)	1.07*
P126	20	7:13	1:2 (3:1)	17.1

*Asterisks indicate that the segregation obtained does not significantly deviate from the expected (Mendelian) ratio according to the *Chi*-square test at the significance level of 0.05

**Table 10:**
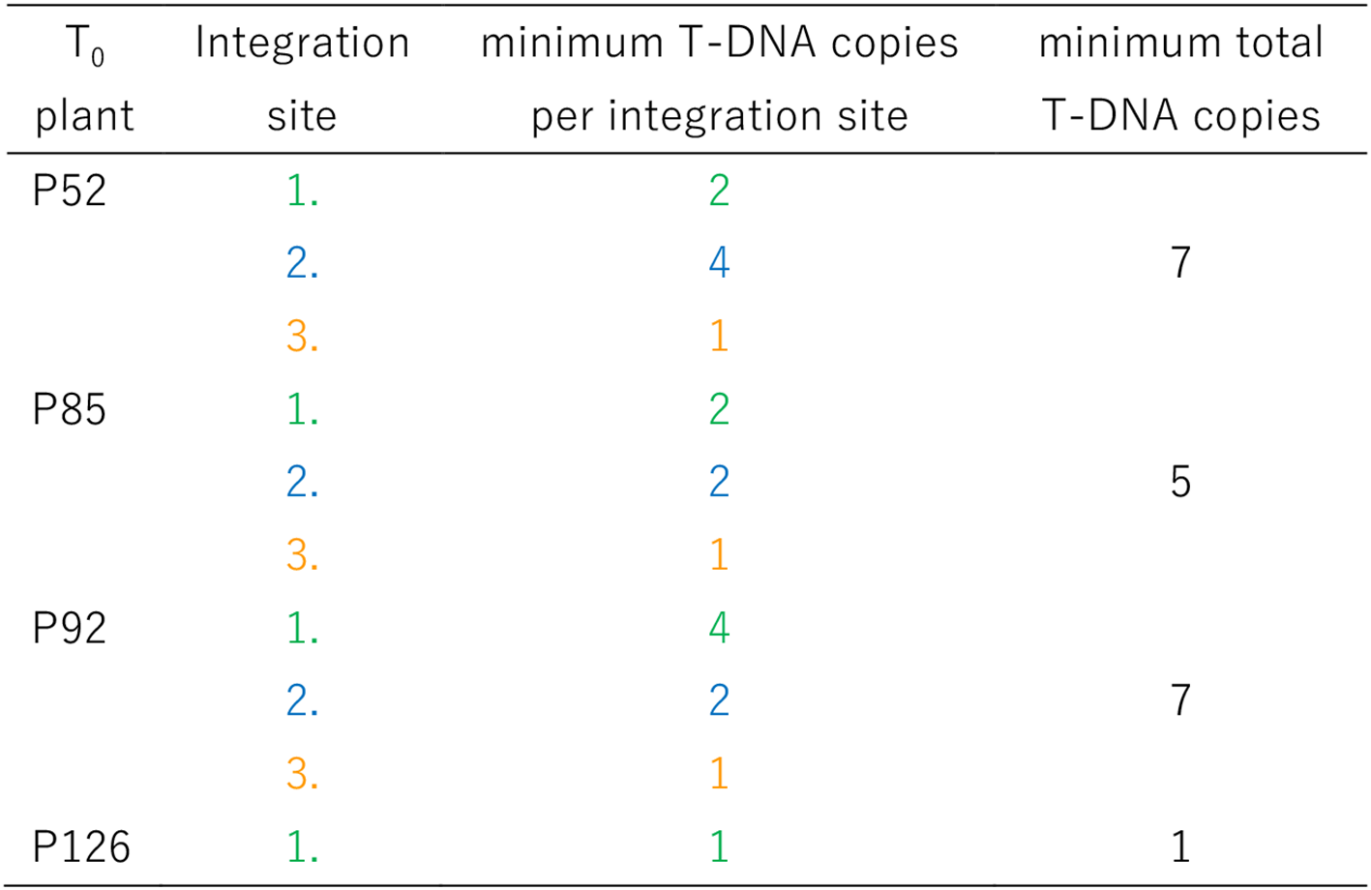
Genomic integration sites and co-segregating (genetically coupled) T-DNA copies according to the DNA gel blot shown in Fig. 7

T-DNA copies belonging to the same integration site are indicated by the color code also used in Figure 7. Owing to potentially overlapping bands and to the limited number of T _1_ siblings analyzed, the numbers of integration sites and/or copy numbers are to be interpreted as minima

#### Genomic integration of transgenes

A DNA gel (Southern) blot analysis was performed to provide unambiguous evidence of stable transgenicity and to determine the numbers of transgene integration loci and of T-DNA copy numbers in individual transgenic plants (Fig. 7; Table 10). Of note, all PCR-positive plants tested by DNA gel blot were confirmed to be transgenic, which indicates a high reliability of the former method, albeit false PCR-positives cannot be entirely ruled out. P92 must had been a chimera, where one T-DNA copy (orange arrowhead) was present only in a plant sector beyond the sample taken for DNA extraction. Consequently, this copy does not show up in the lane for the T_0_ plant, whereas it is present in all P92-derived progeny analyzed.

## Discussion

In the present study, a novel method of genetic transformation of the oilseed crop camelina was established based on agro-inoculation of immature zygotic embryos (Fig. 1). Due to their comparatively high regenerative capacity, immature embryos are the most commonly used explant type for genetic modification of the rather difficult-to-transform monocotyledonous cereals (Kumlehn and Hensel 2009). In dicots, however, there have been only very few examples of the use of immature embryos for transformation, e.g. in *Datura innoxia* [11], soybean [12], and the tree legume *Leucaena leucocephala* [13], because satisfactory transformation efficiencies can usually be achieved more readily with seedling-derived explants in these plants. Previously published work on transgenesis in camelina is largely based on the use of the floral dip method, in which flowers are infiltrated with *Agrobacterium* so that T-DNA can be transferred into egg cells, leading to transmission of the integrated transgenes to embryos of the next-generation seeds [5, 14]. For the use of seedling explants in camelina, only anecdotal results are available without solid evidence of stable transgenicity and inheritability [7–9]. Whilst dipping is advantageous due to its methodological simplicity and independence from in vitro cell cultures, this principle is however also associated with comparatively low and inconsistent transformation efficiency and a genotype dependency which limit its applicability [15]. In addition, dipping is not compatible with the direct transfer of DNA or other biomolecules such as RNAs or proteins. However, in genome editing, especially in the case of higher precision approaches that are typically associated with reduced efficiency, the higher gene dose during the transient expression phase after ballistic transfer of DNA or delivery of non-integrating ribonucleoprotein complexes of Cas endonuclease and guide RNA may be of great advantage.

The recent establishment of efficient plant regeneration from immature zygotic embryos of camelina [10] constituted an essential basis for the present approach. The stimulation of neoplastic development through wounding and the resulting formation of adventitious shoots is a well-known phenomenon in biology and biotechnology [16–18]. In the present study, however, the rather unexpected observation was made that the stimulation of adventitious shoot formation can be drastically enhanced by a combination of wounding and agro-inoculation. Agro-inoculation without wounding, on the other hand, had no such effect (Table 1). A decrease in regeneration efficiency can be observed with most *Agrobacterium*-based methods, particularly when sensitive cell cultures are used [19]. On the other hand, we are not aware of any case reported so far, in which agro-inoculation has triggered intensified plant regeneration. However, whether the improvement in adventitious shoot formation observed in the present study is a plant response to the agrobacteria, as assumed, or is simply due to the additional treatment with the culture media used for inoculation and co-culture, remains to be clarified.

Previous studies have demonstrated that pre-culturing explants under suitable conditions prior to agro-infection can enhance transformation efficiency [20, 21]. In our previous work on the establishment of the regeneration method employed here, hypocotyls of immature camelina embryos were examined histologically after cultivation for three days [10]. At that point in time, epidermal and subepidermal cells had already started proliferating, whereby the resultant development of leafy structures later entailed plant regeneration.

The efficiency of selection for transgenicity of the developing shoots depends on various influencing factors. Different plant species and their various explant types can respond very differently to various selection agents and depending on their concentration. A high selection pressure immediately after DNA transfer, for example, can be problematic, as the explants have just undergone the usually severe stress of tissue dissection and DNA transfer. Moreover, the transgenic sectors of the explants must first reach enough abundance of the detoxifying product of the selection marker gene. On the other hand, a relatively high transient expression of transferred DNA that has not been incorporated into the genome in transformed cells can also cause a particularly high resilience to the selection agent and thus significantly support the preferential development of transgenic tissues. The establishment of a suitable selection regime therefore usually requires empirical approaches to optimization when developing new transformation methods. In the present investigation, the application of 50 mg/L hygromycin for two weeks followed by 25 mg/L for another two weeks or the consistent application of 25 mg/L over four weeks proved to be suitable selection regimes for the generation of transgenic plants (Tables 5 and 6). These conditions are within the usual range for plant transformation systems [22]. However, the proportion of non-transgenic regenerates that was consistently above 50% appears to be comparatively high in the experiments carried out here. However, in many studies using other plant species, although comparable in principle, data on this aspect is rarely provided, so that a systematic comparison is hardly possible.

The choice of *Agrobacterium* strain is often decisive for the successful establishment of plant transformation systems [23, 24]. In the present study, only hypervirulent strains were tested, with which comparatively high transformation efficiencies can be achieved due to additional copies of virulence genes [25], whereby AGL1 proved to be more suitable than LBA4404/pSB1 for the method established here in terms of both regeneration and transformation efficiency. By contrast, the conventional *Agrobacterium* strain GV3101 has been mostly used in current transformation systems for *Brassicaceae* species. This also applies to camelina [5, 26], although higher efficiencies have been achieved with the At503 strain in direct comparison with GV3101 [15].

The genetic transformation of camelina using the dipping method has been described in the literature using various genotypes, in particular the varieties Suneson and Calena [6]. A direct comparison of four accessions revealed notable differences in terms of transformation efficiency [15]. We also know from our own experience that there are considerable differences in suitability for the dipping method between genotypes [14]. In the present study, it is demonstrated that the newly established method based on the use of immature zygotic embryos can achieve largely comparable transformation efficiencies across three different genotypes including two current cultivars at a level that is on par with current methods for other *Brassicaceae* species [22, 27].

## Conclusion

In the present work, stable genomic integration and inheritability of transgenes is demonstrated in a convergent manner using herbicide resistance, PCR, DNA gel blot and specific fluorescence of the transgene product GFP. To the best of our knowledge, we are the first ever to analyze transgenic camelina plants using DNA gel blots, providing not only unequivocal evidence of their stable transgenicity, but also exemplary data on T-DNA insertion loci and copy numbers.

In summary, the establishment of a new method for *Agrobacterium*-mediated transformation of camelina using immature zygotic embryos provides a useful alternative to the previously used dipping method, especially due to its low genotype dependence, robustness, and efficiency. Based on the experiments performed, the following conditions deviating from the preliminary standard protocol described in the materials and methods section were identified as particularly suitable: *Agrobacterium* strain AGL1, *Agrobacterium* inoculum with OD_600_ of 0.6, and hygromycin at a concentration of 50 mg/L for two weeks followed by another two weeks at 25 mg/L during explant cultivation on shoot initiation media. The regeneration principle used here offers the prospect of further methodological developments for the direct transfer of DNA or other biomolecules into regenerable cells, resulting in improved and new possibilities for genome editing of this agronomically valuable and versatile crop plant.

### Methods

#### Plant material

Experiments were conducted with the experimental camelina (*Camelina sativa* (L.) Crantz) line Cam139. Where specifically stated, the cultivars Calena (provided by the IPK Genebank) and Ligena (Deutsche Saatveredelung AG, Lippstadt, Germany) were used. Donor plants were grown as previously described [10] in glasshouse chambers under natural daylight supplemented with 16-hour illumination by high-pressure sodium lamps, providing an additional light intensity of 300 to 400 µmol m^-2^ s^-1^. Day and night temperatures were adjusted to 20 and 18 °C, respectively, and the relative humidity to approximately 65%. The plants were regularly watered with a 1% solution of fertilizer Hakaphos Blau (15% nitrogen, 10% phosphorus, 15% potassium; Compo Expert, Germany).

#### Transformation vector and bacterial strains

**Fig. 8:**
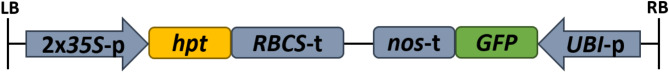
Schematic representation of the T-DNA region of the binary vector pGH429. LB, left T-DNA border; *UBI*p, *UBIQUITIN 4 − 2* promoter from parsley (*Petroselinum crispum*); *GFP*, enhanced green fluorescent protein-coding sequence; *nos*-t, *nopaline synthase* terminator of *Agrobacterium tumefaciens*; *RBCS*-t, *ribulose-1.5-bisphosphate carboxylase small subunit E9* terminator of pea (*Pisum sativum*); *hpt*, *hygromycin phosphotransferase* of *E. coli*; 2 × *35 S*-p, doubled-enhanced *CaMV35S* promoter; RB, right T-DNA border

Transformation experiments were conducted using a reporter gene construct allowing for the visual detection of transformants. For cloning of the *Petroselinum crispum UBIQUITIN 4 − 2* promoter (*PcUBI*p) [28], polymerase chain reaction (PCR) was conducted with primers GH-PcUbi-F1 (5’-GATACCGTCGACGTCAAAAATTACGGATATGAATATAGGC-3’) and GH-PcUbi-R1 (5’-CTTGCTCACCATGGCGCTGCACATACATAACATATC-3’) using the plasmid pDE_CAS9 [29] as template. The amplicon was integrated into the pGFP-Amp vector [30] linearized by NcoI via Gibson assembly generating plasmid pGH302. The whole expression cassette was then moved via SfiI into plasmid p6i-2 × 35 S-E9t (DNA-Cloning-Service, Hamburg, Germany) to generate pGH429. This binary vector was introduced into the *Agrobacterium tumefaciens* strains AGL1 [31] and LBA4404/pSB1 [25] using the electroporation method described by Lin [32]. The functional elements contained in the T-DNA of this binary vector are depicted in Fig. 8.

#### Preparation of the bacterial inoculation medium

The *Agrobacterium* strain LBA4404/pSB1 was grown overnight at 28 °C, 180 rpm in CPY medium (0.1% yeast extract, 0.5% peptone, 0.5% sucrose, 8 mM MgSO_4_, pH 7.0), 100 mg/mL spectinomycin and 10 mg/mL tetracyclin. Where specifically stated in the results section, AGL1 was used for comparison. This strain was grown in MG/L medium containing 5 g/L mannitol, 1 g/L L-glutamic acid, 250 mg/L KH_2_PO_4_, 100 mg/L MgSO_4_, 100 mg/L NaCl, 5 g/L tryptone, 2.5 g/L yeast extract, and 1 µg/L biotin [33], with the addition of rifampicin (10 mg/L) and carbenicillin (100 mg/L). The cultures were adjusted to OD_600_ of 0.2 with an Ultrospec 10 Cell Density Meter (Amersham Biosciences, UK), and glycerol stocks (200 µL with 15% glycerol) were prepared and stored at -80 °C for further use. 100 µL of the agrobacterial glycerol stock was plated onto solid CPY medium (1.2% Micro Agar, Duchefa Biochemie, Netherlands) containing the appropriate antibiotics and grown in the dark at 21 °C. After two to three days of *Agrobacterium* growth, colonies were scraped from the plate using a spatula (Cell Scraper, Corning Science Mexico), and then were resuspended in inoculation medium to obtain an OD_600_ of 0.3, if not stated otherwise.

#### Explant and nutrient media preparation

Immature zygotic embryos were dissected according to a previously described method for plant regeneration based on hypocotyl-derived adventitious shoot formation [10]. More specifically, embryos of the mid-walking stick stage (1.2–1.6 mm) were excised from immature silicles 12 to 14 days after pollination (Fig. 1a, b and c). The embryo explants were pre-cultivated for three days, if not stated otherwise (for standard culture media see Table 11). After pre-cultivation, the explants were carefully pricked at 3 to 5 positions using a sterile hypodermic needle (0.3 mm x 25 mm; BD Precision Glide TM, USA) (Fig. 1) under a binocular microscope (Zeiss Stemi 2000, Germany), if not stated otherwise. To prevent desiccation, the wounded explants were immediately transferred to liquid inoculation medium in 6-well plates with 20 explants per well (CellStar, Greiner Bio-One, Germany).

**Table 11:** Composition of standard culture media used to generate Transgenic camelina plants

Components	Pre-culture medium	Inoculation medium	Co-cultivation medium	Shoot initiation medium	Shoot elongation medium	Rooting medium
MS minerals, NH_4_NO_3_-free	1x	1x	1x	1x	1x	0.5x
NH_4_NO_3_ (mM)	5	5	5	5	5	2.5
B5 vitamins	1x	1x	1x	1x	1x	1x
Sucrose (g/L)	30	30	30	30	20	10
IAA (mg/L)	2	-	2	2	0.1	0.1
Acetosyringone (µM)	-	150	150	-	-	-
Hygromycin (mg/L)	-	-	-	2 weeks 25	-	-
Timentin (mg/L)	-	-	-	150	150	150
Phytagel (g/L)	4	-	4	4	4	4
pH	5.7	5.7	5.7	5.7	5.7	5.7

Among the experiments conducted to optimize and validate the transformation method established in this study, the effects of pre-cultivation period and time point of pricking on the formation of transgenic plants were comparatively investigated.

#### Agro-inoculation and co-cultivation of immature zygotic embryos

The *Agrobacterium* suspension was shaken at 100 rpm for 15 to 30 min at room temperature before inoculation. The inoculation medium was completely removed from the 6-well plate containing the immature embryos and 3 mL of *Agrobacterium* suspension was added per well. During the initial step of inoculation, the plate was placed in a desiccator, and a vacuum of 450 mbar was applied for 3 min (Fig. 1e). The vacuum system consisted of a vacuum pump (Diaphragm Pump, VACUUBRAND, Wertheim, Germany) to which a desiccator was attached. After switching off the pump, air was slowly let into the desiccator over a period of 60 s to minimize tissue damage. After a 12-minutes resting period without vacuum, the *Agrobacterium* suspension was removed, and the explants were briefly blotted onto filter disks. Afterwards, the inoculated embryos were co-cultivated in the dark at 21 °C for 48 h on co-cultivation medium (Table 11), if not stated otherwise. Culture conditions were maintained according to the protocol described in Rezaeva et al. (2024). Accordingly, cultures were kept overnight in the dark at 24 °C, followed by incubation at 25 °C with a 16-hour photoperiod, using cool-white fluorescent lamps with a photon flux density of 60 µmol m − 2 s − 1.

#### Formation of adventitious shoots and plant regeneration

Following co-cultivation, explants were distributed to Petri dishes (2 cm height, 14.5 cm diameter) containing shoot initiation medium (Table 11) using 20 explants per dish. From this point until transfer to soil substrate, the cultures were incubated at 24 °C at night and 25 °C during the day. The illumination time with cool-white fluorescent lamps was 16 h per day and the photon flux density was 60 µmol m^− 2^ s^− 1^. After two weeks, the plates were renewed for another 2 weeks of culture. Then, the developed adventitious leafy structures and shoots were separated from the original explants (Fig. 1) and placed on shoot elongation medium (Table 11) for two weeks. Afterwards, the shoots formed were cultivated in vent containers (Duchefa Biochemie, Haarlem, Netherlands) with rooting medium (Table 11) for four weeks. Finally, plantlets were transferred to soil substrate for further development, analysis, and generative propagation. Among the experiments conducted to optimize and validate the transformation method established in this study, the effects of pricking and agro-inoculation as well as of their combination on adventitious shoot formation were comparatively investigated.

#### Screening for the presence of the GFP transgene

Genomic DNA was isolated from the leaves of young camelina plants (2 weeks after sowing) using a phenol chloroform-based procedure [34]. Genomic DNA of non-transformed plants was used as a negative control. For PCR analysis, the *GFP* gene was amplified (541 bp) using the following primers to confirm the presence of the transgene in regenerated plants: forward primer 5′-GGTCACGAACTCCAGCAGGA-3′, reverse primer 5′-TACGGCAAGCTGACCCTGAA-3′. The PCR conditions were set as follows: 95 °C for 5 min, followed by 40 cycles at 95 °C for 30 s, 59 °C for 30 s, 72 °C for 1 min, and a final extension step at 72 °C for 7 min. The amplified PCR products were visualized and photographed using electrophoresis with MIDORI^green^ Xtra staining (Nippon Genetics Europe, Düren, Germany). The results were documented using the UVP GelStudio from Analytik Jena GmbH (Jena, Germany), equipped with a FastGene^®^ Blue/Green LED Transilluminator DE (Nippon Genetics Europe, Düren, Germany).

#### Generation of T1 plants and segregation analysis

To identify an optimal hygromycin concentration for the selective germination of transgenic progeny, T_1_ seeds were germinated on media with various hygromycin concentrations (2.5, 5, 10, 20, 40 and 80 mg/L). These media consisted of 50% MS minerals [35] supplemented with 1% (w/v) sucrose and were solidified with 0.4% (w/v) Phytagel. Hygromycin was added after autoclaving, and then the medium was filled into square plates (120 × 120 × 17 mm, Greiner Bio-One, Germany). Twenty seeds were sown per plate and were then incubated under a 16/8 h (light/dark) photoperiod at 24 °C.

The identification of resistant and susceptible seedlings was performed after one week of germination using 40 mg/L hygromycin. For segregation analyses based on specific GFP fluorescence, seeds were germinated on hygromycin-free medium. The fluorescence was monitored as described previously [36] applying LED laser monochromatic excitation at 488 nm and image capture using a Canon EOS M200 camera equipped with a 520-nm long-wave pass filter. Canon Digital Photo Professional 4 software was used to convert the images taken in RAW format to TIF or JPG [36]. In addition, the roots of seedlings grown on hygromycin-free medium for four days were examined for GFP fluorescence using a confocal laser-scanning microscope (LSM 780; Zeiss, Germany) equipped with a 10X/0.45 M27 objective. GFP was excited with a 488-nm argon laser and the emitted light was detected within the range of 493–598 nm wavelength. For segregation analysis using PCR, seeds of primary transgenic camelina plants were germinated in soil. Genomic DNA was extracted from two-week old seedlings and examined using *GFP*-specific primers as described above for primary transgenics.

#### DNA gel blot

The *GFP* hybridization probe for the DNA gel (Southern) blot was generated using the same primers as for the PCR analysis of plantlets. The amplicons were labeled with digoxygenin (PCR DIG Probe Synthesis Kit, Roche Diagnostics, Mannheim, Germany). Genomic DNA samples (25 µg) from PCR-positive T_0_, T_1_ and non-transgenic control plants were digested with HindIII, separated by electrophoresis through an 0.8% agarose gel, and transferred to a positively charged nylon membrane (Roche Diagnostics) following the manufacturer’s instructions. Each blot was hybridized with the *GFP* probe. Signal detection and probe stripping were carried out following the DIG Application Guide for Filter Hybridization (Roche Diagnostics, Mannheim, Germany).

#### Statistical data analysis

The transformation efficiency was determined as percentage of PCR-proven transgenic plantlets related to the number of *Agrobacterium*-inoculated explants. All statistical analyses were conducted utilizing the SigmaPlot 14.0 software package (Systat Software, Inpixon, Palo Alto, CA, USA). For optimization experiments, Normality (Shapiro-Wilk) test and Equal Variance (Brown-Forsythe) test were conducted. Provided all data complied with both of these preconditions, all pairwise comparisons of treatments were performed via Tukey test. Where only individual pairs of treatments to be compared fulfilled the requirement of normal distribution and equal variance, their comparison was carried out using Student’s t-test. In the case of normal distribution of data that, however, did not comply with equal variance, Welch’s t-test was performed. Data not complying with normal distribution were subjected to non-parametric Kruskal-Wallis analysis of variance on ranks, followed by pairwise comparisons of treatments using Student-Newman-Keuls method. Segregation of progeny of primary transgenic plants was evaluated by comparison of observed with expected ratios of transgenic and non-transgenic siblings using the *Chi*-square test.

## Data Availability

No datasets were generated or analysed during the current study.
